# Simulation of the song motor pathway in birds: A single neuron initiates a chain of events that produces birdsong with realistic spectra properties

**DOI:** 10.1371/journal.pone.0200998

**Published:** 2018-10-05

**Authors:** Cristiano Giordani, Hector Rivera-Gutierrez, Sun Zhe, Ruggero Micheletto

**Affiliations:** 1 Instituto de Fisica, Universidad de Antioquia, Calle 70 No. 52-21, Medellin, Colombia; 2 Instituto de Biologia, Universidad de Antioquia, Calle 70 No. 52-21, Medellin, Colombia; 3 Computational Engineering Applications Unit, Head Office for Information Systems and Cybersecurity, RIKEN, 2-1 Hirosawa, Wako-shi, Saitama, Japan; 4 Riken Brain Science Institute, 2-1 Hirosawa, Wako-shi, Saitama, Japan; 5 Yokohama City University, 22-2 Seto, Kanazawa Ward, Yokohama, Japan; Georgia State University, UNITED STATES

## Abstract

Birdsong is a complex learned behavior regulated by Neuromuscular coordination of different muscle sets necessary for producing relevant sounds. We developed a heterogeneous and stochastically connected neural network representing the pathway from the high vocal center (HVC) to the robust nucleus of the arcopallium (RA) neurons that drive the muscles to generate sounds. We show that a single active neuron is sufficient to initiate a chain of spiking events that results to excite the entire network system. The network could synthesize realistic bird sounds spectra, with spontaneous generation of intermittent sound bursts typical of birdsong (song syllables). This study confirms experiments on animals and on humans, where results have shown that single neurons are responsible for the activation of complex behavior or are associated with high-level perception events.

## Introduction

Birds use acoustic signals for communication purposes and birdsong is known to play a prominent role in sexual selection by influencing female preferences and for territorial defense [1]. This is a very complex behavior regulated by different hormonal and neural mechanisms that interact in an intricate and hierarchical way [1–3]. Birdsong production has been studied for a long time and it is important as a small-scale model of higher level language system. The actual connection circuitry of birds or human brain is complex and very difficult to clarify [4]. Nevertheless, we know that bird brain consists of neural pathways where vocal sounds are produced via phonation through pneumatically induced vibrations [5]. In addition, we learned that birds modulate their vocal tract to filter and produce sounds at different frequencies [3]. Given that the sound generation involves modulation of the vocal tract and the coordination of breathing patterns in such a way that the syrinx receives airflow to vibrate and produce consistent sounds [6, 7], birdsong requires a highly coordinated neuromuscular activity to produce sounds of variability and recognizable details.

Birdsong production involves mainly three different muscle sets: upper vocal tract, syrinx and respiratory system [8]. Previous studies have determined that coordination of these muscle sets for song production, at least in birds that learn their song (passeriformes: oscines, hereafter songbirds), is determined by a group of brain nuclei and their connecting pathways [3, 8]. The one responsible for song production is known as the motor pathway, and this neural architecture comprises the HVC (high vocal center), the robust nucleus of the arcopallium (RA) and the tracheosyringeal motor nucleus [3, 8, 9]. The similarities between human speech and birdsong [10], and the complexity of this learned behavior in birds, have stimulated the study of the neural architecture and processes responsible for birdsong production. By using mathematical models, previous studies have attempted to understand and emulate the spiking neural network that produces birdsong [11, 12]. Notably, the model introduced by Jin in 2007 [13] realized a very detailed but idealized model of sequence generation for birdsong simulation. In that study, neurons are statically connected by predetermined sequences and neuronal theory is based on Hodgkin-Huxley [14] model. Our study improves those works that use deterministic connections, by applying stochastic rules to connect neurons realizing random connection with defined statistic, as in real brains [15, 16]. In previous models, for each class of neurons used, units were considered identical to each other. Here neurons are realized with the required realistic heterogeneity implementing the biologically plausible Izhikevich model [17, 18] for simulation. All these improvement and modernization of previous studies resulted in a persuasive evidence that complex behavior like sound generation in birds (a paradigm of language in superior animals) can be initiated by a single neuron that drives a small sized network pathway, supporting many outstanding experiments on humans and animals [19–22].

## 1 Methods

We use a realistic and biological plausible network model (an Izhikevich spiking network [18]) to reproduce the neurological pathway from the HVC to the robust nucleus of the arcopallium (RA) that drives muscle tension for the bird labial oscillation [15, 16]. These neurons are connected to realize sequential spiking (see for example Hahnloser, 2002 or Spiro, 1999) [23, 24]. There could be infinite methods to realize sequential spiking and stereotypical firing that results in the characteristic acoustical features of birdsong. Neurons could be linearly connected in a feed forward manner (for example Abarbanel, 2004) [12], or we could realize other *ad hoc* circuitry that produces firing sequences. However, a rigid and geometric sequential neuron-to-neuron connection scheme is not realistic for a biological system where neuronal connections are known only by their topological statistics [23, 25]. Moreover, neurons characteristics must be heterogeneous (neurons of the same type are actually different from each other) and excitatory and inhibitory neurons ratio should be biologically plausible [18, 26]

In our tests neuronal connection structure is random (as neural connections are in real brains) but probability of connection to the robust nucleus of the arcopallium is increased in sequence to realize randomness and nevertheless maintain an increasing excitation flow to the RA cells. Results are random for each simulation that is repeated and averaged over several tests for reproducible outputs. The constant activity of one single neuron appears to be the causal entity that drives the complex behavior of the neural system that generates synthetic sounds. The sounds are studied and analyzed in their spectral characteristics, but also we converted them to digital waveform in “.wav” format. These synthetic songs are in this way playable with common digital means for audible sounds and those can be compared with real birdsongs. Files are given in the supplemental material (S1 Audio: “tEvol_PLOS.wave”). We simulated the neural stream that emulates the HVC up to the RA. An object, implemented in the programming language *python*, represents each neuron. We use a very efficient type of *quadratic integrate and fire* neuron model, developed by Izhikevich [18]. In this model the two main variables taken into account are the intracellular membrane voltage *v* and the cell recovery potential *u* as in the following:

$$\begin{array}{ccc} \frac{dv}{dt} & = & k_{1}v^{2}+k_{2}v+k_{3}-u+I\\ \frac{du}{dt} & = & a(bv-u)\\ \text{if}v\geq 30mV & \text{then} & v\leftarrow c,u\leftarrow u+d \end{array}$$ (1)

The variables *I*, *v* and *u* represent current, voltage and recovery potentials. The three parameters *k*_1_, *k*_2_ and *k*_3_ are obtained by fitting the dynamics of real cortical neurons, they are set to 0.04, 5 and 140 respectively. With these parameters choice *v* results to be scaled in mV and time in mSec (we will use *mV*, *mA* and *mSec* units in our results, however the reader should be aware that, due to variables substitution implicit in the Izhikevich model, biologically realistic values could be different) [17, 26]. The other four parameters *a*, *b*, *c* and *d* define the dynamical behavior of the model.

Heterogeneity is introduced by adding a random variable in the parameter generation algorithms. For the excitatory neurons *a* = 0.02, *b* = 0.2, *c* = −50 + 10*x^2^ and *d* = 2 − 1*x^2^, for the inhibitory ones *a* = 0.02 + 0.08*x, *b* = 0.25 − 0.05*x^2^, *c* = −50 and *d* = 2. In this equation *x* is a random variable between 0 and 1 (*x*^2^ represents simply a different random variable). With this scheme baseline values are added to random fluctuating components for the heterogeneity. An initialization procedure generates a number of HVC neurons and the same amount of RAs, for each of these groups 80% are excitatory and 20% inhibitory.

Our parameter choices are based on Izhikevich 2003 and realize neurons of “chattering spiking” (CH) types [26].

Connections to neighbor neurons are defined by an array of integers $\bar{A}$ that points to neurons labels and by an array of floats $\bar{W}$ that contains the corresponding connections strength values (from 0 to 1).

Neurons belonging to the same type (HVC or RA) are connected with each other with a modified small world architecture [27, 28] of dimension one. This means that each neuron has two neighbors, one on its left and one on its right. No random connections are implemented within the same neuron type group, randomness is realized by heterogeneity and random connections between groups as explained below. A single neuron in the HVC group, called *initiator* and labeled as number one, initiates the spiking, and because of this connection structure, all HVC neurons are set to spike in sequence accordingly to random connections of increased strength as described hereafter.

In previous work of Abarbanel et al. [12] HVC neurons are all identical and connected in an exact sequence to drive the RAs network. This type of neuronal sequence is not stochastic so biologically implausible [25]. The statistical connection algorithm that realizes randomness defines the connection strength *W*_*ij*_ for two neurons i and j is

$$\begin{array}{c} \forall \;RA_{j}\;\;,\;\;\forall \;HVC_{i}\;\;,\;\;if\left(\frac{i}{N_{p}}\right)\geq X\{\begin{array}{c} true:\;W_{ij}=h_{min}+X^{\prime }\left(h_{max}-h_{min}\right)\\ false:W_{ij}=0 \end{array} \end{array}$$ (2)

where *i* and *j* are the neurons index for the HVC (*i*) and the RAs (*j*) groups. *N*_*p*_ is the total number of RAs, *X* and *X*′ are random numbers between 0 and 1. For every neuron in HVC, this function generates an uniform random number *X* in the range between 0 and 1. This value is compared with $\frac{i}{N_{p}}$; if the latter is greater than *X*, the neuron is connected to the corresponding *RA*_*j*_. In this way, when the ratio $p=\frac{i}{N_{p}}$ grows the probability of connection does as well and reaches 100% when *i* = *N*_*p*_. The connection strength *W*_*ij*_ is a random value ranging from a minimum value of *h*_*min*_ = 0.5 to *h*_*max*_ = 1; in case of no connection *W*_*ij*_ = 0.

So, for each neuron in the network, the input is determined adding up all the contributions coming from connected pre-synaptic neurons. The parameters *W*_*ij*_ are used as conductance weights. In other words, the current *I* in Eq 1 is

$$\begin{array}{c} I_{i}=\Sigma_{j}W_{ij}*v_{i} \end{array}$$ (3)

here the index *i* of *I*_*i*_ and *v*_*i*_ represents the current neuron and the index *j* the neurons connected to it.

**Fig 1:**
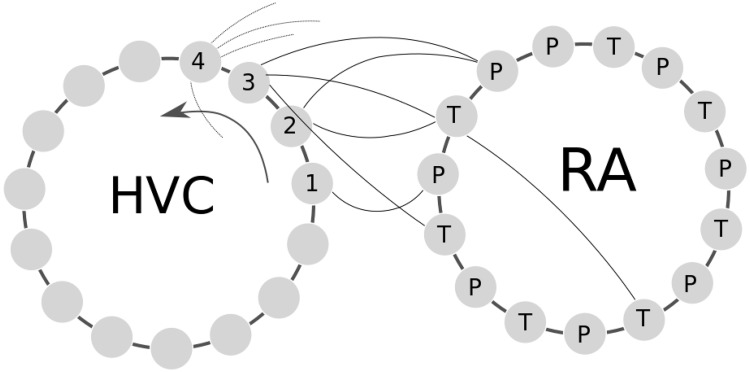
A graphical representation of the neural network described. The two rings are HVC and RA neurons connected with a simplified small world (S.W.) network structure of dimension one. The left ring represents a number of HVC neurons connected to their adjacent neighbors. In a similar fashion are setup the RA neurons in the adjacent ring on the right. To elucidate the HVC-RA stochastic connection algorithm, the first few HVC neuron are schematically numbered as “1”, “2”, “3” etc. and the random connections to RAs neurons are represented by lines going on the right. The probability of connection to RA grows counterclockwise (arrow) to realize the sequential spiking. Connections are unidirectional from HVC to RA and are indicated by the curved lines (not all connections are shown, those from neuron 4 onwards are interrupted for clarity, connections are random and differ for each simulation). Each neuron is a python object that executes the Izhikevich neural model (Eq 1). Half of RA neurons connect to muscles that drive the bronchial pressure (P) and half to the labial tension (T). A neuronal recruitment model is used to integrate neuronal spikes and activate muscles (Eqs 4 and 5). RA neurons connect to a physical model of the syrinx regulated in this way by tension and bronchial pressure (Eq 6).

When this neuronal architecture is constructed (see Fig 1 for a sketch of it), the single initiator neuron can start a chain of events that brings the HVC network to spike and induces, sequentially, an increasing number of RA neurons to spike. This structure realizes both biologically plausible randomness/heterogeneity and strong stereotyped firing observed in birdsong [23].

Parts of the RA neurons are connected to the muscle that regulates the syringeal tension and parts to those that regulate bronchial pressure [12]. We chose at random 50% of RA being of the first type and the remaining RA neurons of the other. The selection is done using the count number of each of them and selecting odd and even numbers for the two classes. At any given time, the tension and pressure driving input of the syringeal and bronchial muscular apparatus are function of the membrane potentials accordingly to a muscular recruitment process. In other words, the tension of the muscles is proportional to the number of firing motor neurons (recruitment [12]) and decays exponentially if there are no firing ones, or if the total potential is lower than a threshold. This is realized by the following equation:

$$\begin{array}{cc} \frac{dT}{dt} & =+pos\{\Sigma_{t}\left(Ra_{t}/N-Th\right)\}-T/\tau_{t}\\ \frac{dP}{dt} & =+pos\{\Sigma_{p}\left(Ra_{p}/N-Th\right)\}-P/\tau_{p} \end{array}$$ (4)

where *pos* is the positive function, *pos*(*x*) = *x* if *x* is positive, zero otherwise. *Th* is a threshold fixed at -64 mV, RA_*t*_ and RA_*p*_ are the membrane potentials for the two RA populations (*t* and *p* are the corresponding indexes and N is the total number of neurons for each population. In our case of the two groups *Ra*_*t*_ and *Ra*_*p*_ have same size, that is half of the total RA neurons). The values *τ*_*t*_ and *τ*_*p*_ are the time constants of the recruiting process, also those are supposed to be equal for the two populations *τ*_*t*_ = *τ*_*p*_ = 10 msec. To calibrate suitable variables range, two scaling functions are defined:

$$\begin{array}{cc} \alpha (T) & =\alpha_{1}T+\alpha_{0}\\ \beta (P) & =\beta_{1}P+\beta_{0} \end{array}$$ (5)

these are proportional to the time-dependent values for tension and pressure (T and P). In our case *alpha*_0_ = 0.9, *alpha*_1_ = 50*10^−3^, *β*_0_ = 15*10^−3^ and *β*_1_ = 8.75*10^−3^. These constants were determined analyzing the equations in the work of Abarbanel [12] and adapting to our specific neural network. The differential equation describing the physical motion is as follows [12, 29]:

$$\begin{array}{ccc} \frac{dx}{dt} & = & y\\ \frac{dy}{dt} & = & y-\alpha \left(T\right)x-Cx^{2}y+\beta \left(P\right)y \end{array}$$ (6)

The variable *x* represents the difference between the position of the labium (prephonatory site) and its rest point, *y* represents the corresponding velocity. These formalisms may look counter intuitive, but we used these conventions to keep the same expression format of previous well-known literature (for example again Abarbanel 2004).

In this equation the coefficient of y represents the linear dissipation, in this case the variable pressure *β*. The pressure variability is caused by the neural recruiting process and it acts on the velocity *y* damping its amplitude. The coefficient *C* instead is the quadratic non-linear factor of this model. Using the works of Gardner and one of Laje [11, 29] as a guideline, and considering our different scaling variables, this is set to C = 0.4. Preliminary tests (not shown) indicated that this value keeps main peak centered about 500 Hz. It is a critical value that is kept constant in all the simulations presented here for reason of uniformity between all graphs.

**Fig 2:**
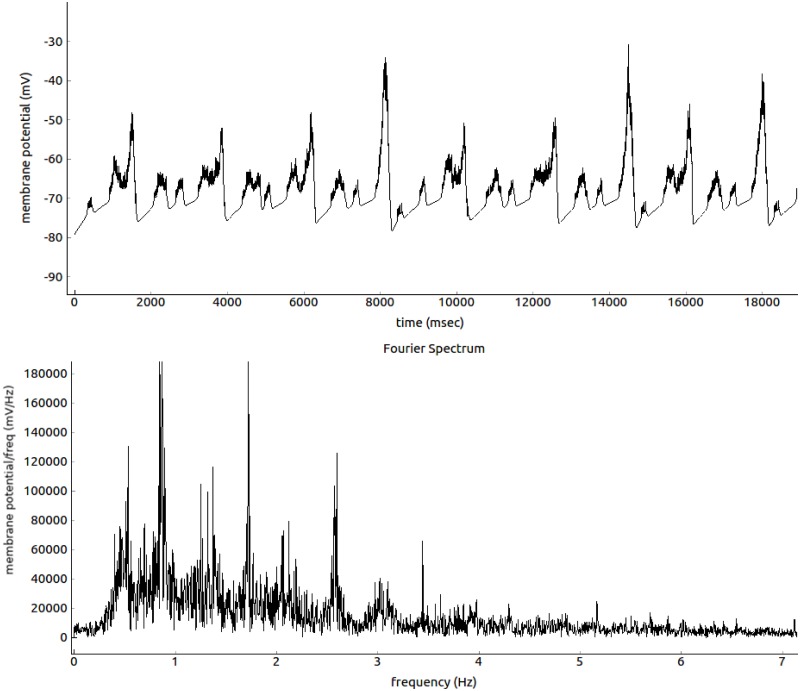
Top panel: the average membrane potential for a group of 20 HVC neurons (connected as in Fig 1) in function of time (each point is 1 msec). Bottom panel: the Fourier transformation of the same membrane potential. Strong low frequencies delta waves are prominent, peaks at higher frequencies in the *α* waves range are also noticeable.

**Fig 3:**
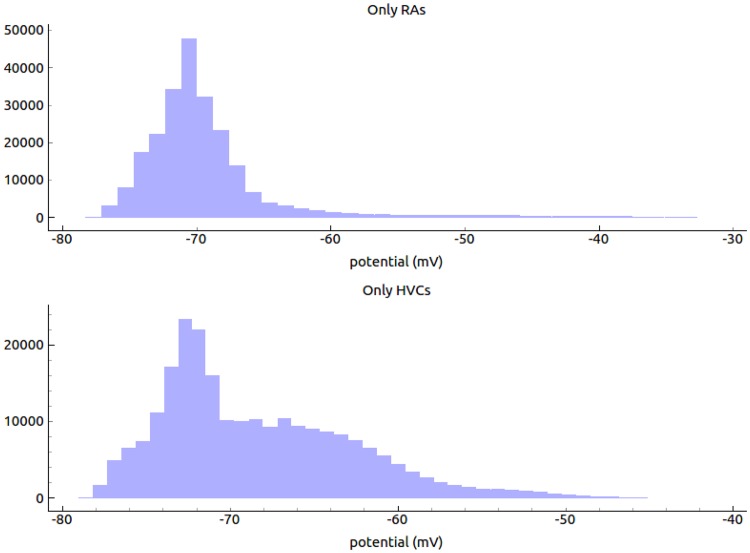
Distribution of the membrane potential. Top panel: The membrane potential distribution calculated for 20 RAs cells, these cells activity is sharper and spread in a narrower range compared to the HVCs cells. Bottom panel: the same distribution for the HVCs cells group. Activity is sparse over a wider range of potential values. Parameter of the simulation are as in Fig 5.

We integrate this differential equation with the Euler method. This method has been chosen since it is fast and precise enough. In literature simulation with Izhikevich neurons are integrated with time step of *dT* = 1msec [18], here we use *dT* = 0.1 msec for better stability and robustness to noise. A python controlled graphic engine (blender, www.blender.org) was used to change color and vertical length of each neuronal block when active and was used to visualize in quasi real-time the dynamics of the network for testing, optimizing and evaluation (an example of these visualizations is shown in the supporting materials S1 Video: “blendNeurVisual.avi”). A statistical characterization of the resulting membrane potential is shown in Figs 2 and 3.

## 2 Results and discussion

**Fig 4:**
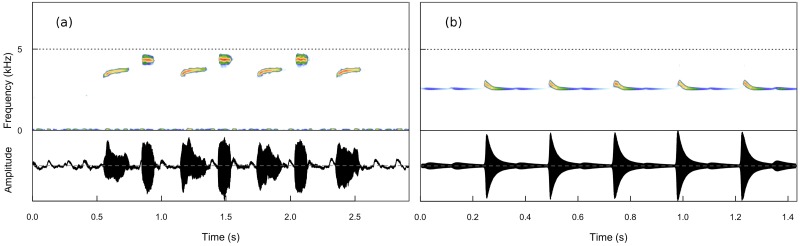
Panel (a): two notes song of a common songbird, the great tit Parus major. In panel (b) the resulting sounds from our model is shown for comparison. The sounds produced by the very simple neural network described here, resemble real birdsong in intermittent behavior, tonality, syllable duration and fundamental frequency. An example wave file representing the sounds generated by our model is in the supporting media S1 Audio: “tEvol_PLOS.wave”. Simulation parameters in this plot are those of Fig 5 and sampling rate increased of a factor of three for easier visualization and comparison.

Birds produce a large diversity of sounds, which usually include pure-tone whistles, broadband sounds, frequency and amplitude modulations, click-like sounds, and noise. In despite of the large diversity in bird sounds, birdsong is recognized as tonal [30]. Given that birdsong production is regulated by respiratory patterns [31], songs have different duration, with an average of two seconds. In addition, a note (the minimum phonetic unit of songs) can last between 10 and 100 ms. Although the fundamental frequency of birdsong usually lies between 3 to 5 KHz [30], birds may produce songs in a large range of frequencies. Our synthetically produced sounds resemble birdsong in tonality, duration, and fundamental frequency. In Fig 4, we present a simple two notes song of a common songbird, the great tit Parus major, (Fig 4a), compared to one of the resulting sounds from our model (Fig 4b). Considering the fact that the neural pathway simulated in the model is extremely simple and triggered by a single activating neuron, the similarities between the real sounds and the simulated one are striking.

Our simulation results are compatible with those obtained by Henry D. I. Abarbanel et al. [12] that realized a model of the song motor pathway in birds. We have significantly improved that model by introducing the Izhikevich equations, describing the variations over the time of the voltage response and the recovery variable which represents the difference between all inward and outward voltage-gated currents [18].

Moreover, different from the work mentioned above where the number of neurons were fixed to 10 units, our model allows more neurons, theoretically unlimited quantity, and was constructed by establishing a biological plausible stochastic architecture between the nucleus HVC and the robust nucleus of the arcopallium (RA). For each simulation run used in our work only one HVC type neuron, named *initiator* above, is stimulated with always the same constant strength (except when the influence of current is studied) and it induces the HVC small world network to become active and to drive the second group of RAs neurons as described previously. Eqs (1), (4), (5) and (6) are integrated with a time step of 0.1 ms for a total simulation time of 1 second. The nonlinear parameter C (equal to 0.4) and the two recruitment time constants *τ*_*t*_ and *τ*_*p*_ are kept both equal to 10 ms for the entire sets of simulations (except when tau was examined). The parameters *τ* represent the decay rate of the pressure and tension variables, in other words how fast they decay once stimuli are removed. To test reproducibility and stability, each simulation run has been repeated 3 times with different random seeds when generating neuronal parameters and connection strengths. So different runs differ slightly one from another. Trend and behavior were identical for all simulations, plots shown are one example of the three simulations, not an average of them.

**Fig 5:**
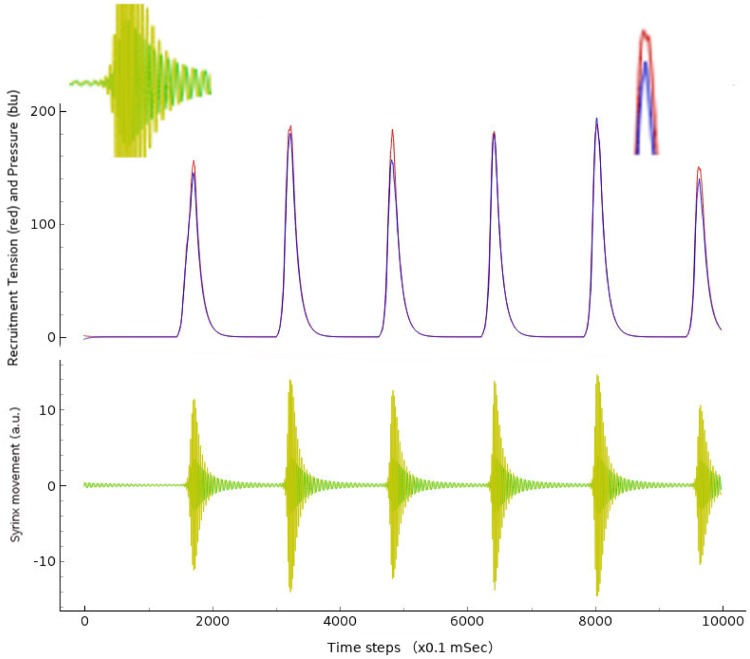
A simulation of the spiking neural network model described. Burst of oscillations produce spikes of pressure and tension (upper panel) that consequently generate oscillation of the syrinx (lower panel). Top panel: recruitment tension and pressure are shown in red and blue, respectively. Bottom panel: bird syrinx movement in X and Y axes are shown in green and orange, respectively. The insets on the top left and top right represent a zoom-up of the first burst in XY and pressure/tension respectively. Simulation settings: C = 0.4, neurons number = 20, tau = 10 ms, INLVL = 0; I current = 10mA. The horizontal axis represents the integration time steps *dT* = 0.1ms, so total duration of simulation is one second.

Results of the simulation show intermittent burst of oscillation, which scaled in time, will result in the birdsong-like syllables. In Fig (5), we can observe the wave of pressure and tension the network produces in the upper panel, whereas in the lowest the actual labial movement is shown with clear oscillatory behavior. The network reproduces long period of labial inactivity spontaneously. Notably, one single initiator neuron is the cause of the chain of spikes that finally result in these trains of vibrations that resemble the natural birdsong as reported in many birdsong studies [1, 6].

**Fig 6:**
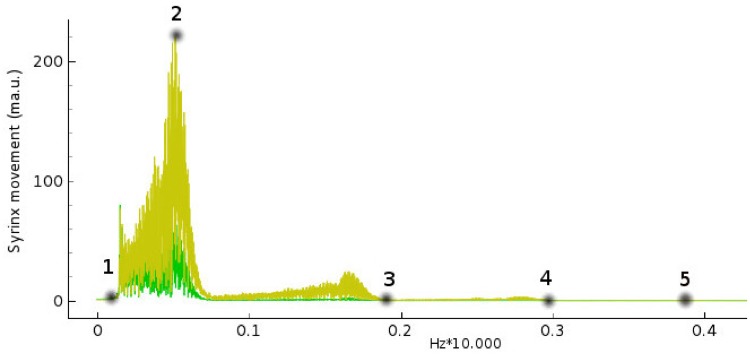
The labeled positions of relevant frequencies in the birdsong spectrum as Fourier transformed from Fig 5. Peak numbered 1 represents the minimal frequency (“Min Freq”) at which the sound begins, number 2 (“Peak 1”) the actual main peak of the sound, number 3 (“Peak 2”) frequency at which the sound collapse, number 4 (“Peak 3”) is the second high-frequency position and number 5 (“Peak 4”) the higher frequency position that characterizes all the sounds produced by the model and represented in Figs 7 to 10. Bird syrinx movement in X and Y axes are shown in green and orange, respectively. The horizontal axis scale is 10^4^Hz, so 0.05 corresponds to 500Hz (every time step corresponds to *dT* = 0.1 ms). The highest peaks 4 and 5 are in some cases not discernible and will be omitted in the plots.

**Fig 7:**
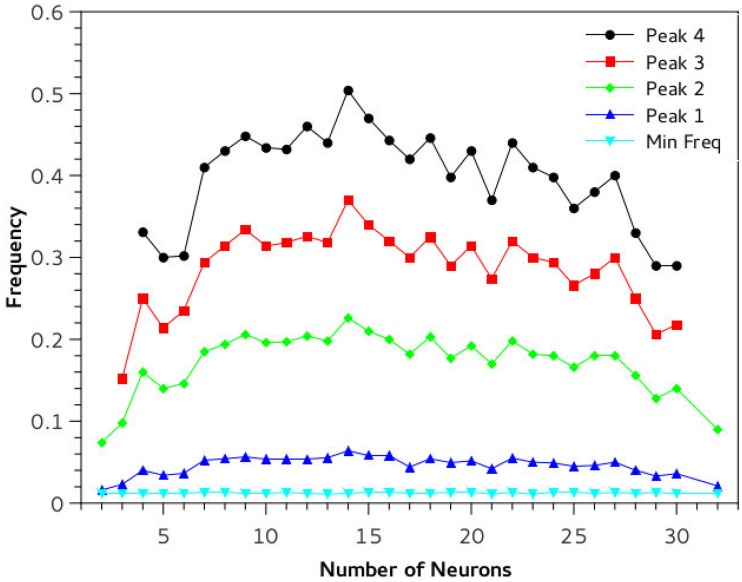
The spectrum characteristics of the complex sound generated by the network in function of network size. Interestingly the central frequency peak of the birdsong increases with network size then have a plateau at about 15-20 neurons where the frequency is more than double than for lower or higher number of neurons. This is a resonant phenomenon that, we hypothesize, could be a model of spontaneous self-regulating mechanisms of higher complexity in real brains. Vertical axis is Hz*10000 and horizontal the number of neurons in the network. Experimental settings: C = 0.4, tau = 10 ms, INLVL = 0; I current = 10mA.

**Fig 8:**
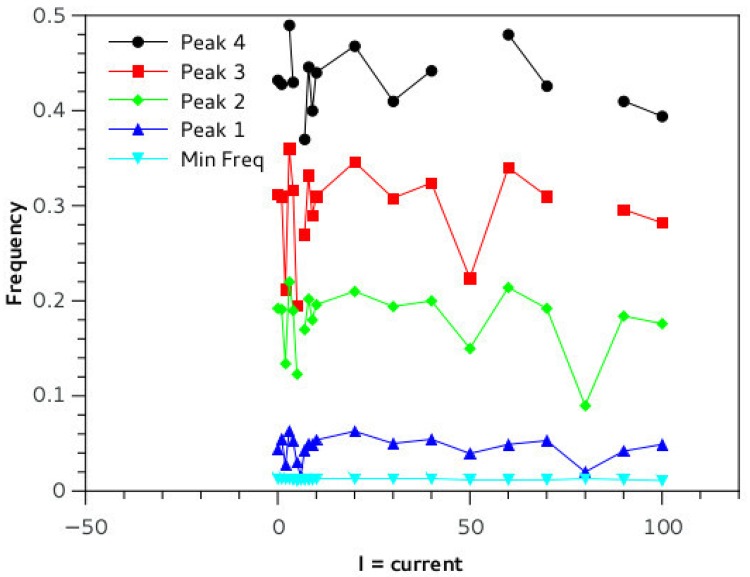
The spectrum of the oscillation of the syrinx is studied in function of the input current of the single neuron that initiates the cascade of spiking in the network. The current level affects the neuron spike rate and a resonant phenomenon is observable around 50 and 80 mA. We can speculate that Birdbrains may modulate the emission frequency by intervening on this current and this would explain variability of birdsong frequencies. Experimental settings: C = 0.4, neurons number = 20, tau = 10 ms, INLVL = 0.

**Fig 9:**
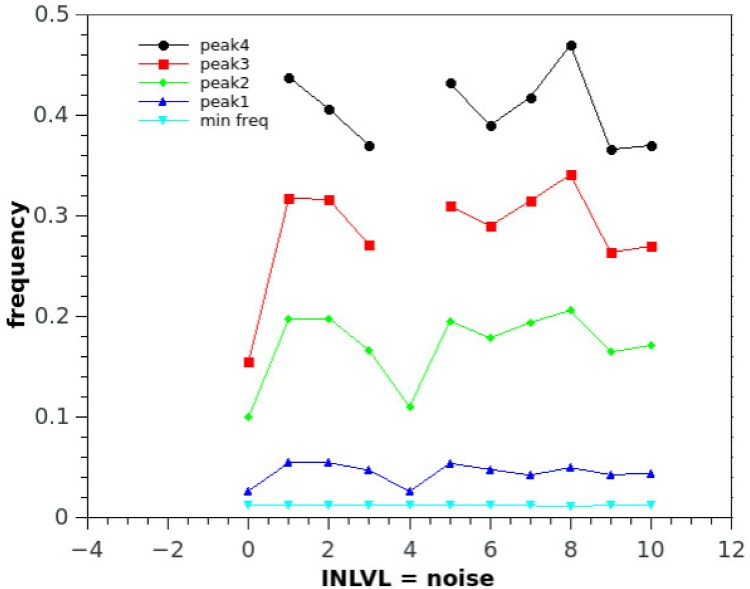
The spectrum of the sound generated by the model is a function of internal noise present in the network. The main peak seems to remain in the order of 500-600 Hz, however central values show a resonant opposite trend (negative peaks around INLVL = 4). Noise is expressed in mV and frequency in 10^4^Hz, as in previous plots. Experimental settings: C = 0.4, neurons number = 20, tau = 10 ms, I current = 10mA.

**Fig 10:**
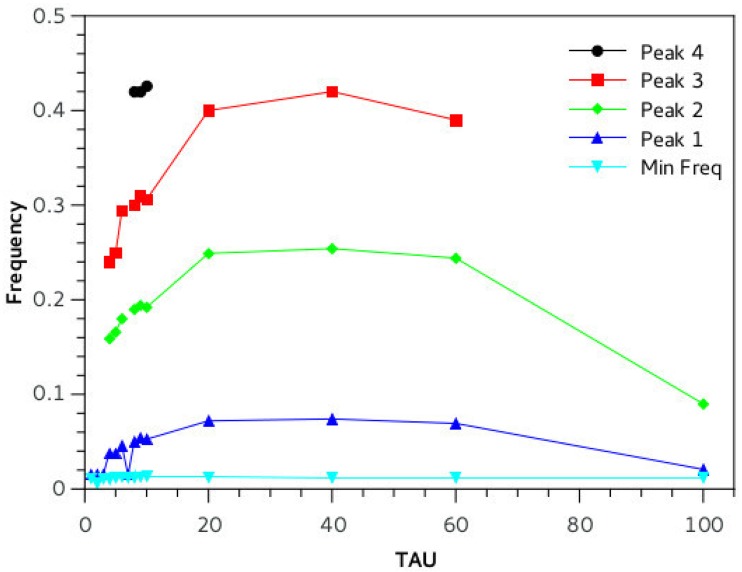
Here we manipulate the physical parameters that describe the decay of bronchial pressure and muscle tension in the physical model of the syringeal and bronchial apparatus (Eq 4). The model results to be robust to variation of *τ*, since there are no major changes in most of the range used (20 to 60 ms). This supports the idea that the labial response is driven mainly by the neural pathway and weakly influenced by the physical properties of the syringeal system. Experimental settings: C = 0.4, neurons number = 20, INLVL = 0; I current = 10mA, *τ*_*p*_ = *τ*_*t*_ for simplicity.

Waveforms so obtained are scaled in time, using a sampling frequency Φ derived directly by the time step of the Izhikevich model integration: Φ = 1/10^−3^ *dT* where dT is the time step and 10^−3^ is a time unit conversion to fit Izhikevich time scale of one millisecond. In the online material it is possible to download a playable file of the waveform obtained (supporting information S1 Audio: “tEvol_PLOS.wave”, generated by the *scipy.io.wavfile.write* library with sampling rate of Φ and scaled over 16 bits). Beside acoustical analysis, a comparative frequency study of the output of the simulation has been performed. The Fourier spectrum of the wave is taken and prominent peaks and frequency positions are studied in function of relevant network parameters (Fig 6). Neural networks systems are extremely non-linear, so definition of parameters is fundamental for stable and reproducible performance of the network. We conducted a series of simulations changing a variety of parameters keeping C constant at 0.4: we varied HVC and RA type neurons number from 1 to 32 (for each set of neurons) (Fig 7). The main pitch of the generated sound is represented by peak number 2, and the position more than doubles in value with increased network size, for example for N = 5, peak 1 frequency is about 100 Hz, whereas for N = 15 it reaches about 500 Hz. Interestingly, once the network is big enough, about 20 neurons, frequency stabilizes and then drops as there is a mechanism that realizes an optimal network size for the system. The amplitude of the input stimulus given to the *initiator* neuron is studied in the range 0 to 100 mA, Fig 8. The network is affected by a generalized noise level, a current value that is superimposed homogeneously on all neurons of the entire network to emulate real brains internally generated noise (this parameter is added to *I*_*i*_ of Eq 3). We call it here INLVL (internal noise level) and it is examined from 0 to 10 mA. Also in this case, noise produce resonant falls in the spectrum, as shown in Fig 9. Eq 4 describe the threshold phenomena occurring in the neurological process of *recruiting*. $-\frac{T}{\tau_{t}}$ represents the exponential decay from the maximum recruiting value reached. The speed of decay is *τ*_*t*_ or *τ*_*p*_ depending on the different variable in consideration (bronchial pressure or muscle tension). In our simulations these are chosen to be both equal and to range between few to 100 msec (Fig 10).

## 3 Conclusions

We constructed a random and heterogeneous spiking network with a realistic neural model to emulate the HVC to RA motor pathway in birds. Our model is made by few tens of interconnected neurons per class, it is a minimal model based on current literature concerning the structure of bird brains. Given the limited knowledge about the exact neuronal connections, this neural pathway is purely theoretical. Moreover, the size and level of interconnections is minimal compared to real brains. Even with these limitations, the simulation demonstrates that it is possible to build a simple neuronal pathway that realizes complex sounds strikingly similar to birdsongs. It shows that a single neuronal activation can initiate the chain of events that leads to the song generation.

We could demonstrate that a recruiting system is at play during sound generation and that synthetic waveforms comparable to birdsounds are produced by the network. Not only sounds are similar in center tonality and frequency profile, but also train of syllables are generated spontaneously by the network with timing and rhythm compatible to natural birdsongs. Our sounds are similar to those described by Nowicki as typical birdsong, which includes nearly sinusoidal pure tones and harmonic sounds, where frequency and amplitude modulations may be present [32]. Moreover, birdsong frequency usually ranges between 3 and 5 KHz, but birds may sing between 100 Hz and above 10 KHz [32] and our sounds, using the simulation time in the Izhikevich neuronal model, results in raw sounds centered around 500-600 Hz. The waveforms obtained could be scaled in frequency by mere re-sampling and sounds similar to birdsongs of species with higher tones can be obtained, see the supplemental material S1 Audio for an example audio file of our waveforms (“tEvol_PLOS.wave”).

The chain of spiking that produces this complex behavior, is generated by a single initiator neuron that is spiking first, all other spiking in the network are spontaneous consequence of this single neuron activity, in accordance to recent experiments [33]. Our model and results are consistent with the idea that the expression of complex behaviors can be generated by a statistically connected chain of neurons similarly to previous experiments on animals and models. This study suggests the existence of a simple, self-regulating and extremely efficient mechanism for the generation of complex activity as birdsongs and paves the way for the complete simulation of more complex animal behavior with simple and primitive spiking networks instruments.

## Supporting information

S1 Audio An example of the sounds produced by our network. Sounds are similar in center tonality and frequency profile, train of syllables are generated spontaneously with timing and rhythm compatible to real birdsongs. Birdsong frequency usually ranges between 3 and 5 KHz, but birds may sing between 100 Hz and above 10 KHz. The waveforms produced by the network could be scaled in frequency by mere re-sampling to obtain sounds similar to those of species with birdsong of higher tones.
(WAVE)

S1 Video A video that shows how we visualize the dynamics of our network. Each block represents a neuron, the hight of the block is proportional to the membrane potential and can be seen changing in time. Connections between neurons are not shown, but the two rings represent the HVC neurons and the RA ones. The central block represent the syrinx muscle system and its elongation is proportional the air pressure. This visualization system was useful especially at the beginning of our research as simple *sanity check*, but also was useful to have a qualitative insight on the flow of information along the neural pathway.
(AVI)
